# Integrated Multiomics Analysis Reveals Manual Therapy Restores Autophagic Homeostasis via the AMPK/ULK1 Axis in Chronic Low Back Pain

**DOI:** 10.1155/prm/6688692

**Published:** 2026-08-26

**Authors:** Liangyu Xie, Zuozhen Yin, Litao Shao, Jiale Ma, Haowen Cheng, Liang Shi, Gongchang Yu, Shengnan Cao, Weidong Su, Bin Shi

**Affiliations:** ^1^ School of Medical Information and Artificial Intelligence, Shandong First Medical University & Shandong Academy of Medical Sciences, Jinan 250117, China, sdfmu.edu.cn; ^2^ Shandong Key Laboratory of Biomechanics, Neck-Shoulder and Lumbocrural Pain Hospital of Shandong First Medical University, Shandong First Medical University & Shandong Academy of Medical Sciences, Jinan 250062, China, sdfmu.edu.cn; ^3^ Innovation Institute of Integrated Traditional Chinese and Western Medicine, Shandong First Medical University & Shandong Academy of Medical Sciences, Jinan 250117, China, sdfmu.edu.cn

**Keywords:** AMPK/ULK1, chronic low back pain, manual therapy, metabolomics, proteomics

## Abstract

**Background:**

Chronic low back pain (CLBP) is characterized by persistent neuroinflammation and metabolic dysregulation, extending beyond local tissue injury to central neural maladaptation. While manual therapy (MT) is a clinically validated intervention, its capacity to modulate the neuro‐immune‐metabolic axis remains poorly understood.

**Methods:**

We established a complete Freund’s adjuvant (CFA)–induced CLBP rat model and used a combination of in vivo behavioral and molecular techniques to study MT intervention mechanisms. The therapeutic effects were evaluated via multidimensional approaches combining behavioral assays, histology, and high‐throughput dual‐tissue (prefrontal cortex and multifidus muscle) proteomics and metabolomics.

**Results:**

MT intervention significantly attenuated mechanical hyperalgesia and suppressed pro‐inflammatory cytokines (IL‐6, IL‐1β, and TNF‐α) in muscle tissues. Integrated omics revealed distinct reprogramming of metabolic pathways, including GABAergic synapses and oxidative phosphorylation. Crucially, the CLBP model exhibited aberrant upregulation of autophagy initiation markers (AMPK, ULK1, and LC3B) coupled with substrate accumulation (p62), indicative of pathological autophagic stress. MT partially normalized the altered expression of autophagy‐related proteins.

**Conclusion:**

Our findings from a CFA‐induced inflammatory multifidus injury rat model suggest that AMPK/ULK1‐linked autophagy signaling may correlate with the therapeutic effects of MT on inflammatory muscle‐type low back pain; these preclinical observations require further verification in heterogeneous clinical CLBP cohorts and multiple animal models.

## 1. Introduction

Chronic low back pain (CLBP) represents a pervasive global health challenge, affecting approximately 21%–48% of the adult population and serving as a leading cause of disability worldwide [1, 2]. CLBP can be categorized into chronic inflammatory pain and neuropathic pain. Persistent pain has been shown to cause significant physical and mental health impacts for 23% of individuals [3, 4]. Regarding the economic burden of musculoskeletal disorders (MSDs), CLBP imposes the heaviest toll on global welfare losses, accounting for 43% of the total economic burden of MSDs and equivalent to 0.61% of the global gross domestic product [5]. Clinical guidelines and expert consensus statements on CLBP have been widely implemented over the years [6]. Treatment methods for CLBP are generally classified into pharmacological and nonpharmacological approaches [7]. Pharmacological treatments primarily alleviate pain by modulating neuronal activity, but their therapeutic efficacy is limited and they carry significant toxicity and dose‐limiting side effects [8, 9]. Manual therapy (MT) can block pain signal transmission pathways and produce analgesic effects by altering neural plasticity [10]. MT remains the most commonly used nonpharmacological treatment for CLBP [11]. Current pathophysiological models posit that CLBP involves a failure of inflammation resolution. Persistent production of cytokines such as tumor necrosis factor‐alpha (TNF‐α) and Interleukin‐6 (IL‐6) sensitizes peripheral nociceptors and drives central sensitization in brain regions like the prefrontal cortex (PFC). While the analgesic effects of MT are well documented, the black box” of its molecular mechanism limits its clinical optimization. Emerging evidence in mechano‐immunology suggests that mechanical forces can be transduced into intracellular biochemical signals that regulate macrophage polarization and cytokine secretion, potentially serving as a nonpharmacological anti‐inflammatory strategy.

Pain may arise through top‐down mechanisms or bottom‐up pathways [12, 13]. Long‐term chronic pain can cause changes in brain regions associated with cognition, attention, sensory processing, and emotional regulation [14, 15]. When brain networks become disrupted or their coordination is altered, it significantly impairs a patient’s ability to process pain‐related information [16, 17].

The PFC, as the center for higher‐order neural activity, plays a crucial regulatory role in pain perception and constitutes a vital component of the brain’s pain cortex [18–20]. Research has sought potential targets for chronic pain using protein datasets from the PFC [21]. CLBP typically originates from dysfunction of the multifidus muscles [22–24]. Multifidus muscle injury compromises spinal stability, potentially leading to increased biomechanical stress and pain signals. It promotes the development of central sensitization, perpetuating chronic pain states [25].

Complete Freund’s adjuvant (CFA) can induce cell‐mediated immunity and act as an immunostimulant to promote immunoglobulin production [26]. It is a commonly used reagent for establishing animal models of CLBP. In this study, a rat model of chronic inflammatory low back pain was established by injecting CFA into the lumbar multifidus muscle. Research on MT for pain treatment has been extensively validated [27]. MT can improve the inflammatory response to pain in localized tissues, enhance the body’s motor function, and reduce the use of analgesic medications [28]. Recent research has found that MT enhances neural connectivity between the peripheral motor network and the PFC [29–31]. However, the molecular mechanisms underlying therapeutic interventions on the brain remain unclear.

Metabolomics provides a powerful analytical approach for elucidating disease‐related metabolic disorders by analyzing alterations in endogenous metabolite levels. Proteomics enables comprehensive characterization of the protein composition, expression patterns, and interaction networks within biological systems [32]. When these omics approaches are integrated, they provide a solid foundation for elucidating the molecular mechanisms by which MT exerts its effects across a variety of pathological conditions. In recent years, advances in multi‐omics integration strategies have provided comprehensive and in‐depth insights into disease‐related molecular networks, facilitating the identification of potential biomarkers and therapeutic targets [33]. This study systematically evaluated the effects of MT on protein expression in the PFC and multifidus muscle, as well as on the metabolite profile in a rat model of CLBP, through proteomics and metabolomics analysis.

Accumulating evidence indicates autophagy acts as a vital effector mediating cellular mechanotransduction under physical loading [34–36]. It operates as a recycling mechanism, disposing of unneeded cytoplasmic parts and organelles during specific physiological and pathological conditions [37, 38]. Current research has demonstrated that MT can alleviate knee osteoarthritis by modulating the PI3K/AKT/mTOR pathway [39] and can ameliorate skeletal muscle damage by regulating autophagy [40]. Adenosine Monophosphate Activated Protein Kinase (AMPK) serves as a sensor for cellular energy status and is crucial for regulating autophagy. AMPK promotes autophagy by directly phosphorylating and activating the ULK1 kinase complex, a key initiator of the autophagy process [37, 41]. AMPK plays a crucial role in chronic inflammatory pain and neuropathic pain. Given that mechanical stimulation can activate AMPK in various cell types, we reasoned that AMPK/ULK1‐linked autophagy may correlate with phenotypic improvements only in this CFA rat model, with direct causal evidence still lacking. However, whether this mechanism operates concurrently in both injured muscle tissue and central pain pathways remains unknown.

The study uses an integrated multi‐omics strategy, combining proteomics and metabolomics, to map the molecular landscape of MT intervention in a rat model of CFA‐induced CLBP. By simultaneously analyzing the proteomic and metabolic profiles of the injured multifidus muscle (peripheral target) and the PFC (central target), we aim to provide a comprehensive system‐level understanding of MT efficacy. Guided by the traditional Chinese medicine (TCM) therapeutic principle of “treating external manifestations to regulate internal physiology,” this study constitutes the first preclinical investigation to dissect the molecular mechanisms of MT from combined central and peripheral dimensions using a CFA‐induced inflammatory multifidus injury rat model. It offers preliminary mechanistic evidence and testable hypotheses to support future clinical validation focusing on inflammatory low back pain subtypes.

## 2. Materials and Methods

### 2.1. Experimental Animals and Groups

**FIGURE 1:**
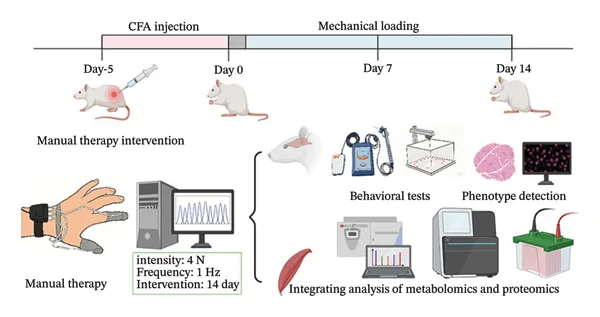
Schematic diagram illustrating the experimental design and workflow of the study. The timeline includes model establishment (CFA injection), manual therapy intervention, behavioral testing (MPWT and OFT), and sample collection for multi‐omics and molecular analysis.

Male Sprague‐Dawley rats (weight, 220–240 g; age, 6–8 weeks) were purchased from Pengyue Laboratory Animal Breeding Co., Ltd (Jinan, Shandong, China). All animals were housed under standard laboratory conditions (22°C–27°C, 50%–70% indoor humidity). Only rats with intact motor function and normal baseline pain thresholds were included; rats with infection or spontaneous pain were excluded. Rats with > 10% weight fluctuation during acclimation, anesthesia intolerance or abnormal gait were also excluded and replaced with spare littermates to keep *n* = 8 per group. Rats were randomized via computer‐generated random numbers, and group assignments were sealed in opaque envelopes for allocation concealment, with 8 animals assigned to the Control, Model, and Treatment groups, respectively. Sample size was predetermined by G∗Power analysis to achieve adequate statistical power for behavioral readouts. All behavioral, histological, and molecular analyses were completed by researchers blinded to group identity. The animal studies received approval from the Animal Ethics Committee at the Institute of Neck‐Pain Hospital of Shoulder and Lumbocrural Shandong First Medical University, approval ID 20250213, and experiments followed ARRIVE 2.0 guidelines. The study design is summarized in Figure 1.

### 2.2. Establishment of the CLBP Model

The rat model of chronic inflammatory low back pain was established via intramuscular injection of CFA, following a modified protocol previously described by Tokunaga et al. Rats were anesthetized via inhalation of 2%–3% isoflurane (RWD Life Science, Shenzhen, China) in oxygen flow. After shaving and disinfecting the dorsal lumbar region, the L4–L6 vertebrae were identified by palpation. Chronic inflammation was induced by a bilateral injection of CFA (*Mycobacterium tuberculosis*, Sigma‐Aldrich, St. Louis, MO, USA) into the lumbar multifidus muscle at a dosage of 50 μL per site. The Control group received an equivalent volume of sterile saline injection to control for the stress of the injection procedure.

### 2.3. MT Intervention

To simulate clinical MT techniques with high reproducibility, we employed a tactile measurement finger sleeve (Finger TPS, Pressure Profile System, CA, USA). The Treatment group underwent daily sessions of mechanical stimulation for 14 consecutive days. During treatment, rats were gently secured on a fixation platform to minimize restraint stress. The device could maintain a constant stimulation pressure of 4.0 N and frequency of 1 Hz. Each therapeutic session lasted 10 min. The force magnitude and frequency were selected based on previous studies demonstrating therapeutic efficacy without inducing tissue damage. The Control and Model groups underwent identical handling and fixation procedures but without the application of mechanical force, serving as sham‐treatment controls.

### 2.4. Behavioral Assessment of Mechanical Hypersensitivity

Mechanical sensitivity in rats was assessed using an electronic stinger (Ugo Basile, VA, Italy). We acclimated three groups of rats in the test chamber for at least 20 min, initiating the test once exploratory behavior ceased. The next stimulation must be administered at least 20 s after the previous measurement. We obtained the average of six retraction threshold measurements per rat and regarded this as an indicator of mechanical sensitivity.

### 2.5. Open Field Test (OFT)

The OFT placed rats in an enclosed yet open arena (100 cm × 100 cm × 50 cm) with a black floor surface, where their behavior was recorded via a video tracking system. Evaluation parameters included entries in zone, total distance, and mean speed in zone. During testing, the arena was thoroughly cleaned with 75% ethanol to eliminate olfactory cues. This test was conducted before modeling, after modeling, and after intervention to evaluate the effects of intervention on anxiety and locomotor activity.

### 2.6. Sample Collection and Preparation

At the end of the 14‐day intervention period, rats were fasted overnight and deeply anesthetized with 3% sodium pentobarbital (50 mg/kg, i.p.) or via isoflurane inhalation. Euthanasia was subsequently confirmed by cervical dislocation. Tissue samples from the PFC and the lumbar multifidus muscle were rapidly dissected on ice. Each tissue sample was divided into two aliquots: one portion was immediately flash‐frozen in liquid nitrogen and stored at −80°C for proteomics, metabolomics, and Western blot analyses; the other portion was fixed in 4% paraformaldehyde (PFA) for 48 h for histological examination.

### 2.7. Histological and Immunofluorescence (IF) Analysis

Fixed muscle tissues were dehydrated in a graded ethanol series, cleared in xylene, and embedded in paraffin. Serial sections (5 μm thick) were prepared using a rotary microtome.

### 2.8. Histological Staining

Paraffin‐embedded sections were dewaxed and processed for hematoxylin and eosin (H&E) staining to examine tissue architecture and inflammatory cell accumulation. To evaluate muscle fibrosis and collagen deposition, Masson’s Trichrome and Sirius Red staining were performed according to standard protocols. Slides were scanned using a digital slide scanner (VS200, Olympus, Japan), and images were analyzed using Olyvia software (Olympus) or ImageJ (NIH, Bethesda, MD, USA). The area of collagen deposition (blue in Masson’s, red in Sirius Red) was quantified as a percentage of the total tissue area.

### 2.9. IF

For IF analysis, antigen retrieval was performed by microwaving sections in sodium citrate buffer (pH 6.0). Sections were blocked with 10% goat serum for 1 h at room temperature and incubated overnight at 4°C with primary antibodies against Laminin (to delineate muscle fibers). After washing, sections were incubated with fluorophore‐conjugated secondary antibodies (Alexa Fluor 488/594) for 1 h. Nuclei were counterstained with DAPI. The cross‐sectional area (CSA) of myofibers was calculated from Laminin‐stained images.

### 2.10. Immunohistochemistry (IHC)

Paraffin sections were deparaffinized, rehydrated, and treated with 3% hydrogen peroxide to quench endogenous peroxidase activity. Antigen retrieval was performed via heat induction in citrate buffer. Sections were blocked with 5% bovine serum albumin (BSA) and incubated overnight at 4°C with the following primary antibodies: anti‐IL‐6 (1:200, Proteintech, Cat# 21865‐1‐AP), anti‐IL‐1β (1:200, Proteintech, Cat# 16806‐1‐AP), and anti‐TNF‐α (1:200, Proteintech, Cat# 60291‐1‐Ig). A horseradish peroxidase (HRP)‐conjugated secondary antibody detection system (Dako EnVision) was used, followed by diaminobenzidine (DAB) chromogen development. Sections were counterstained with hematoxylin. The percentage of positive staining area was quantified in 10 randomly selected fields per section using Image‐Pro Plus 6.0 software (Media Cybernetics, USA).

### 2.11. Proteomics Analysis

First, frozen tissue was homogenized using RIPA lysis buffer and a protease inhibitor mixture. The sample was then placed in a high‐speed centrifuge and centrifuged at 13,000 rpm for 20 min. Subsequently, the sample was centrifuged again at 13,000 rpm for 20 min. Protein concentration was determined using the BCA assay kit. Proteins were enzymatically digested using trypsin at a ratio of 50:1. The peptide segments were desalted and dissolved in solvent A (0.1% formic acid in water), then separated on a reversed‐phase analytical column using a Vanquish Neo liquid chromatography system (Thermo Fisher Scientific). The chromatographic gradient was performed as follows: 0–7 min, 7.5% B; 8–9 min, 35% B; 9–10 min, 100% B, where solvent B was 0.1% formic acid, 80% acetonitrile, and 20% water, at a flow rate of 0.7 μL/min. The eluted peptides were analyzed using a timsTOF HT mass spectrometer (Bruker) equipped with a captive electrospray ionization source in positive‐ion mode using data‐independent acquisition (DIA). The mass spectrometry parameters were set as follows: capillary voltage, 1.6 kV; dry temperature, 180°C; dry gas flow, 3.2 L/min; mass range, 300–1500 m/z; ion mobility, 0.7–1.3 V s/cm^2^; and collision energy, 20–59 eV.

The DIA raw data were processed using DIA‐NN software against the UniProt Rattus norvegicus reference proteome database (UP000002494, downloaded on February 8, 2025). The search parameters were set as follows: enzyme, trypsin; maximum missed cleavages, 1; fixed modification, carbamidomethyl (C); variable modifications, oxidation (M), acetyl (Protein N‐term). A target‐reverse decoy database strategy was employed to control the false discovery rate (FDR) at 1% at both the peptide‐spectrum match and protein levels. For reliable quantification, proteins with valid expression values in at least 50% of the samples within any group were retained; missing values were imputed using the group mean (for groups with ≥ 50% valid values) or half of the minimum matrix value, followed by log2 transformation and median normalization across all samples. Differentially expressed proteins were identified using unpaired Student′s *t*‐test with the following thresholds: *p* < 0.05, FC ≥ 2.0. To further control the FDR for multiple hypothesis testing, the Benjamini–Hochberg correction was additionally applied, and proteins with an adjusted *p* < 0.05 were considered statistically significant for the final candidate list.

### 2.12. Metabolomics Analysis

Tissue samples were extracted with methanol–water (4:1, v/v) containing internal standards. After centrifugation, 150 μL of supernatant was collected for LC‐MS analysis. Quality control (QC) samples were prepared by pooling equal volumes of all individual sample extracts and were injected periodically throughout the analytical run to monitor system stability. Chromatographic separation was performed on an ACQUITY UPLC HSS T3 column (100 mm × 2.1 mm, 1.8 μm) using a Waters ACQUITY UPLC‐Class plus system at 45°C The mobile phase consisted of 0.1% formic acid in water (A) and acetonitrile (B) at a flow rate of 0.35 mL/min. The gradient elution program was: 0–2 min, 5% B; 2–4 min, 5%–30% B; 4–8 min, 30%–50% B; 8–10 min, 50%–80% B; 10–14 min, 80%–100% B; 14–15 min, 100% B; 15–15.1 min, 100%‐5% B. The injection volume was 5 μL. Mass spectrometry was performed on a Thermo Q‐Exactive high‐resolution mass spectrometer in both positive‐ and negative‐ion modes with the following parameters: spray voltage, 3800 V (positive) and −3200 V (negative); capillary temperature, 320°C; auxiliary gas heater temperature, 350°C; sheath gas flow rate, 35 Arb; auxiliary gas flow rate, 8 Arb; mass range, 70–1050 m/z; full MS resolution, 60,000; and MS/MS resolution, 15,000.

Raw data were processed using XCMS software (version 4.5.1) for peak detection, retention time correction, and integration. Metabolite identification was performed by matching accurate mass, retention time, MS/MS fragmentation, and isotope distribution against HMDB, Lipidmaps (v2.3), METLIN, and an in‐house library (LuMet‐Animal 3.0) containing over 2000 authentic standards with retention time, MS1, and MS2 information. Metabolites were annotated according to the following confidence levels: Level 1, retention time within ±0.3 min of the standard and Fragmentation Score ≥ 45; Level 2, retention time within ±0.3 min and 0 ≤ Fragmentation Score < 45; Level 3, Fragmentation Score ≥ 45 (without retention time match); and Level 4, Fragmentation Score < 45. Only metabolites with a total identification Score ≥ 36 (out of 80) were retained. For data normalization, ion peaks with RSD > 0.3 across QC samples were removed; metabolites with missing values in > 50% of samples within any group were excluded, and the remaining missing values were replaced with half of the minimum positive value, followed by log2 transformation. Differentially expressed metabolites were identified using unpaired Student′s *t*‐test with thresholds of FC ≥ 1.2 and *p* < 0.05. To further control the FDR, the Benjamini–Hochberg FDR correction was additionally applied, and an adjusted *p* < 0.05 was considered statistically significant. VIP values were calculated from the OPLS‐DA model, and metabolites with VIP > 1.0 were prioritized for follow‐up analysis.

### 2.13. Western Blot Analysis

Proteins were extracted from the frozen muscle tissues using RIPA lysis buffer with phenylmethanesulfonyl fluoride (PMSF) and phosphatase inhibitors. The concentration of protein was measured with a BCA Protein Assay Kit. Equal protein samples of 20 μg were subjected to 10% SDS‐PAGE and subsequently transferred onto 0.45‐μm PVDF membranes. The membranes were blocked for 2 h at room temperature with 5% nonfat dry milk in TBST to prevent nonspecific binding. Subsequently, the membranes were incubated overnight at 4°C with primary antibodies against AMPK (1:2000; Proteintech), ULK1 (1:2000; Proteintech), LC3B (1:1000; Proteintech), p62/SQSTM1 (1:1000; Proteintech), ATG3 (1:1000; Proteintech), ATG5 (1:1000; Proteintech), ATG13 (1:1000; Proteintech), and GAPDH (1:2000; Proteintech). After three washes with TBST, the membranes were incubated for an hour at room temperature with HRP‐conjugated goat anti‐rabbit or anti‐mouse IgG secondary antibodies (1:5000). Using an enhanced chemiluminescence (ECL) kit from Millipore, Burlington, MA, USA, the protein bands were visualized and then imaged with a gel imaging system. Band intensity was quantified using ImageJ software (NIH, Bethesda, MD, USA), with GAPDH serving as the internal loading control for normalization.

### 2.14. Statistical and Bioinformatic Analyses

The analysis of data was performed using GraphPad Prism v5.0 and SPSS v24. All quantitative data are presented as mean ± standard deviation (SD). Single‐time‐point intergroup comparisons across three cohorts were assessed via one‐way ANOVA followed by Tukey’s post hoc test. Linear mixed‐effects models were used to analyze longitudinal mechanical withdrawal threshold data to account for within‐animal repeated measurements. For one‐way ANOVA, effect sizes were calculated as Eta squared (*η* ^2^), with values of 0.01, 0.06, and 0.14 considered small, medium, and large effects, respectively. Group differences with *η* ^2^ ≥ 0.14 were considered practically significant. For proteomic and metabolomic datasets, Benjamini–Hochberg FDR correction was applied to control FDR.

## 3. Results

### 3.1. MT Attenuates Mechanical Hypersensitivity and Restores Locomotor Function in a Rat Model of CLBP

**FIGURE 2:**
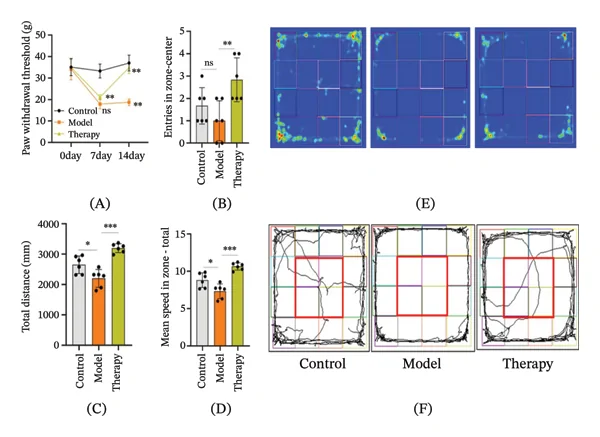
Manual therapy attenuates nociceptive behavior and ameliorates anxiety‐like activity in rats with CFA‐induced CLBP. (A) Time course of the mechanical paw withdrawal threshold (MPWT) measured at baseline (day 0, day 7, and day 14 post‐injection. (B–D) Quantitative analysis of anxiety‐like and locomotor behaviors in the open field test (OFT), including the number of entries into the center zone (B), total distance traveled (C), and average velocity (D). (E, F) Representative motion tracking paths of rats from the Control, Model, and Manual Therapy (MT) groups in the OFT arena. Longitudinal data were analyzed using linear mixed‐effects models with Bonferroni post hoc correction. Data are presented as the mean ± SD. ^∗^ *p* < 0.05, ^∗∗^ *p* < 0.01, ^∗∗∗^ *p* < 0.001, vs. Control group (day 7). ^#^ *p* *< *0.05, ^##^ *p* *< *0.01, ^###^ *p* *< *0.001 vs. Model group (day 14), *n* = 6 per group, ns = no significance (*p* *> 0.05*).

To evaluate the therapeutic efficacy of MT on CLBP, we assessed mechanical sensitivity and spontaneous locomotor behavior. At baseline (Day 0), the mechanical paw withdrawal threshold (MPWT) was comparable across all groups, confirming homogenous baseline sensitivity (Figure 2A). The Model group exhibited a sustained decrease in MPWT relative to the Control group, indicative of mechanical hypersensitivity. On Day 7, while both the Model and Treatment groups showed reduced thresholds compared to Controls, the Treatment group exhibited a significantly higher MPWT than the Model group (*p* < 0.05), suggesting an early analgesic effect. By day 14, the therapeutic benefit was more pronounced; the MPWT in the Treatment group was significantly elevated compared to the Model group and was statistically indistinguishable from the Control group, indicating a complete restoration of mechanical pain thresholds (Figure 2A).

We further investigated the impact of MT on pain‐related functional impairment and anxiety‐like behavior using the OFT. The Model group displayed a marked reduction in exploratory behavior, characterized by decreased total locomotor distance and average speed compared to the Control group. Notably, 14 days of MT intervention significantly reversed these locomotor deficits (Figure 2C,D). Furthermore, assessment of thigmotaxis revealed that the Model group made significantly fewer entries into the Center Zone, a behavior associated with pain‐induced anxiety. MT significantly increased the frequency of Center Zone entries compared to the Model group (Figure 2B), suggesting that MT alleviates both the sensory and affective‐motivational components of CLBP.

### 3.2. MT Reduced Muscle Fibrosis and Improved Functional Recovery of Injured Skeletal Muscle

**FIGURE 3:**
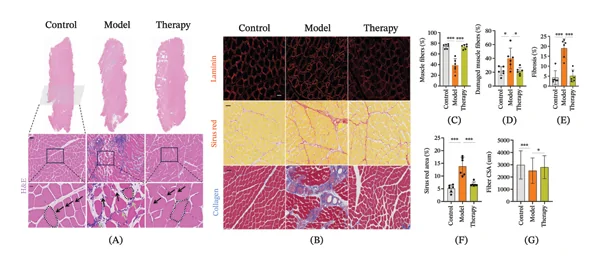
Manual therapy ameliorates histopathological damage and inhibits fibrosis in the multifidus muscle. (A) Representative photomicrographs of muscle tissue sections stained with hematoxylin and eosin (H&E) (magnification 100×). Black arrows indicate pathological features, including inflammatory cell infiltration, disrupted myofiber architecture, and centralized nuclei. Dashed lines delineate the cross‐sectional area (CSA) of individual fibers. (B) Representative histological images of Masson’s Trichrome staining (top row), Sirius Red staining (middle row), and Laminin immunofluorescence (bottom row). In Masson’s staining, muscle fibers are stained red, while collagen fibers (indicating fibrosis) are stained blue. Sirius Red staining visualizes collagen deposition types I and III (red/yellow). Laminin (green) outlines the sarcolemma used for precise CSA quantification. (C) Proportion of morphologically normal muscle fibers; (D) Percentage of damaged muscle fibers; (E) Area fraction of fibrosis quantified from Masson’s Trichrome staining; and (F) Collagen deposition area quantified from Sirius Red staining. (G) Quantification of the mean myofiber cross‐sectional area (CSA) derived from Laminin staining. MT treatment significantly restored fiber dimensions and reduced fibrotic remodeling. Data are presented as mean ± SD (*n* = 6). Statistical comparisons were performed via one‐way ANOVA followed by Tukey’s post hoc test. ^∗^ *p* < 0.05, ^∗∗^ *p* < 0.01, ^∗∗∗^ *p* < 0.001.

To evaluate the therapeutic efficacy of MT protocol on tissue repair, we performed a comprehensive histopathological assessment of the multifidus muscle. H&E staining of the Control group revealed a canonical skeletal muscle architecture, characterized by polygonal myofibers with peripherally located nuclei and a tightly packed fascicular organization (Figure 3A). Conversely, the Model group exhibited profound architectural disarray, evidenced by fragmented myofibers, widened interstitial spaces, and substantial mononuclear cell infiltration—a hallmark of persistent local inflammation (Figure 3A, black arrows). Therapeutic intervention significantly reversed these pathological features. The muscles displayed a restored fascicular geometry and a marked reduction in inflammatory infiltrates, with myofiber morphology closely resembling the uninjured control phenotype.

These morphological observations were corroborated by IF and histochemical assessments of the extracellular matrix (ECM) integrity. Laminin immunolabeling in the Model group visualized discontinuous sarcolemmal boundaries and irregular fiber cross sections, indicative of cytoskeletal disruption (Figure 3B). However, the Treatment group exhibited continuous, well‐defined laminin staining, suggesting the preservation of basal lamina structural integrity. Furthermore, Masson’s trichrome and Sirius Red staining highlighted aberrant collagen accumulation in the Model group, particularly encroaching upon the perimysial and endomysial compartments (Figure 3B). Following the 14‐day MT regimen, the extent of pathological collagen deposition was markedly curtailed, indicating a suppression of the fibrotic response.

Quantitative morphometric analysis reinforced the histological qualitative data (Figures 3C–G). The Model group exhibited a precipitous decline in the proportion of healthy myofibers relative to controls (*p* < 0.001). Conversely, MT intervention significantly prevented this loss, maintaining the healthy fiber population at near‐physiological levels (*p* < 0.001). Parallel quantification of damage markers revealed that both the percentage of disrupted fibers and the total fibrotic area (Figure 3D–F) were elevated in the Model group but were significantly attenuated by MT application. Additionally, the atrophy‐associated reduction in myofiber CSA observed in the Model group was partially ameliorated by the therapy (*p* < 0.05, Figure 3G). Taken together, these data indicated that the MT intervention effectively mitigates injury‐induced fibrosis and facilitates the structural regeneration of skeletal muscle.

### 3.3. MT Promptly Relieved Localized Inflammation in Skeletal Muscle

**FIGURE 4:**
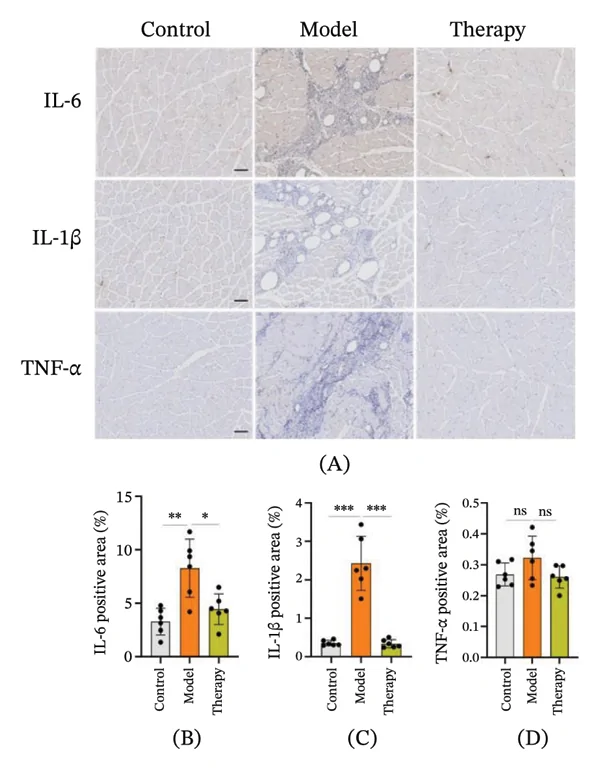
Manual therapy attenuates the expression of pro‐inflammatory cytokines in the multifidus muscle. (A) Representative immunohistochemical staining images showing the expression of IL‐6, IL‐1β, and TNF‐α in muscle tissue sections. Positive expression is visualized as brown staining, while nuclei are counterstained blue. Scale bar: 100 μm. (B–D) Quantitative analysis of the percentage of positive staining areas for IL‐6 (B), IL‐1β (C), and TNF‐α (D). Data are expressed as mean ± SD (*n* = 6). ^∗^ *p* < 0.05, ^∗∗^ *p* < 0.01, ^∗∗∗^ *p* < 0.001.

To determine whether MT influenced local immune reactions in the targeted musculoskeletal regions, we measured the concentrations of the proinflammatory mediators IL‐1β, IL‐6, and TNF‐α. Histological analysis revealed extensive inflammatory cell infiltration within the damaged muscle tissue (Figure 4). Although this cellular influx did not compromise the integrity of muscle fibers, marked upregulation of IL‐1β and IL‐6 expression was detected.

### 3.4. MT Reverses Chronic Pain–Induced Proteomic Alterations in the PFC

**FIGURE 5:**
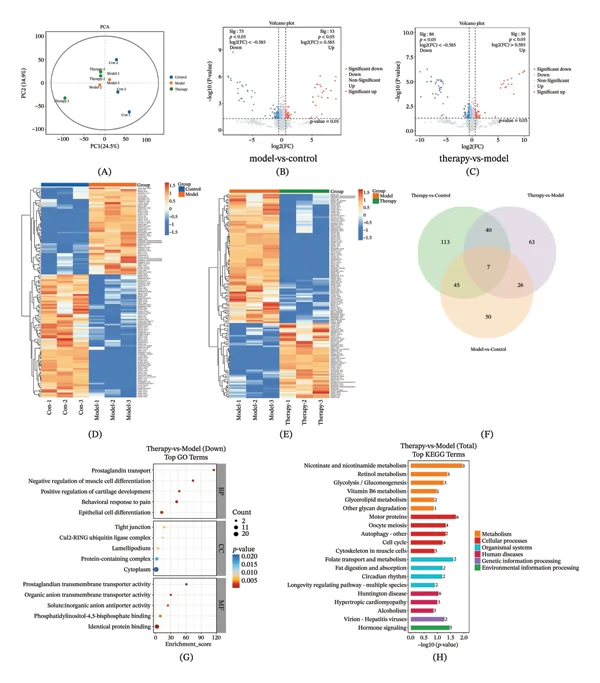
Proteomics analysis of prefrontal cortex tissue of rats. (A) Principal component analysis (PCA) in the Control, Model, and Therapy. (B) Volcano plot of DEPs in the Model and Control groups. (C) Volcano plot of DEPs in the Therapy and Model groups. (D) Heatmap of the expression of DEPs in the Control and Model groups. (E) Heatmap of the expression of DEPs in the Model and Therapy groups. (F) Venn diagram showing the shared and unique identified proteins among the Control, Model, and Therapy groups. DEPs, differentially expressed proteins. (G) GO enrichment analysis of differential proteins. (H) KEGG enrichment analysis of differential proteins.

This study examined protein alterations in PFC tissue samples from rats across different groups. The proteomics results are presented in Figure 5. Principal component analysis (PCA) in Figure 5(A) showed that samples clustered well within the same group, with significant differences observed between groups. A total of 469 differentially expressed proteins were identified across the three groups in this study. Among these, 128 proteins were differentially expressed between the Control and Model groups, with 53 showing an upregulation trend and 75 exhibiting a downregulation trend. The Treatment group and Model group shared 136 differentially expressed proteins, of which 50 showed an upregulation trend and 86 exhibited a downregulation trend (Figure 5B,C). All differentially expressed proteins met the significance threshold of *p* < 0.05. Cluster heatmaps visually displayed the dynamic changes in differentially expressed proteins between the two groups (Figure 5D,E). Subsequently, Venn intersection analysis of significantly differentially expressed proteins revealed 33 proteins (Figure 5F). Among these, 16 proteins exhibited a decrease followed by an increase, while 10 proteins showed an increase followed by a decrease. These proteins represent candidate differential hits that warrant further functional prioritization in future central mechanism studies. GO analysis revealed that the Treatment group and Model group primarily shared similarities in “identical protein binding,” “cytoplasm,” “epithelial cell differentiation,” and “behavioral response to pain” (Figure 5G).

KEGG enrichment analysis revealed a diverse range of annotated pathways, including those related to nicotinate/nicotinamide metabolism, motor proteins, folate transport, vitamin B6 metabolism, and hormone signaling (Figure 5H).

### 3.5. Metabolomics Analysis of the PFC in Rats With CLBP Treated by MT

**FIGURE 6:**
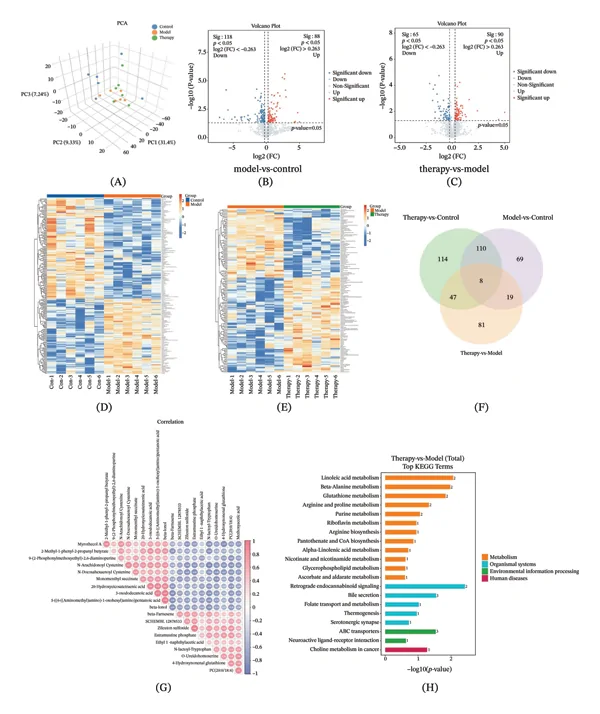
Metabolomics analysis of prefrontal cortex tissue of rats. (A) Principal component analysis (PCA) in the Control, Model, and Therapy. (B) Volcano plot of DEMs in the Model and Control groups. (C) Volcano plot of DEMs in the Therapy and Model groups. (D) Heatmap of the expression of DEMs in the Control and Model groups. (E) Heatmap of the expression of DEMs in the Model and Therapy groups. (F) Venn diagram showing the shared and unique identified proteins among the Control, Model, and Therapy groups. DEMs, differentially expressed metabolites. (G) Pearson’s correlation coefficient analysis shows the metabolic network among significantly different metabolites. (H) KEGG enrichment analysis of metabolites.

Researchers performed metabolomic sequencing on the PFC of rats in each group. PCA revealed significant differences among the three groups (Figure 6A). Volcano plots indicated substantial alterations between the Control group and Model groups, with 206 differentially expressed metabolites detected 88 upregulated and 118 downregulated. Additionally, 155 differential metabolites were identified between the Model and Treatment groups, comprising 90 upregulated and 65 downregulated metabolites (Figure 6B). Cluster heatmaps illustrated the dynamic expression patterns of differential metabolites (Figure 6D,E). Venn diagrams revealed 27 significantly expressed metabolites common to both the Model vs. Control and Model vs. Treatment comparisons. Correlation analysis revealed associations among different metabolites (Figure 6G). KEGG pathway enrichment analysis identified significantly enriched pathways including retrograde endocannabinoid signaling, choline metabolism in cancer, linoleic acid metabolism, and beta‐alanine metabolism—key signaling cascades (Figure 6H).

### 3.6. Proteomics Analysis of Muscle Damage in Rats With CLBP Treated by MT

**FIGURE 7:**
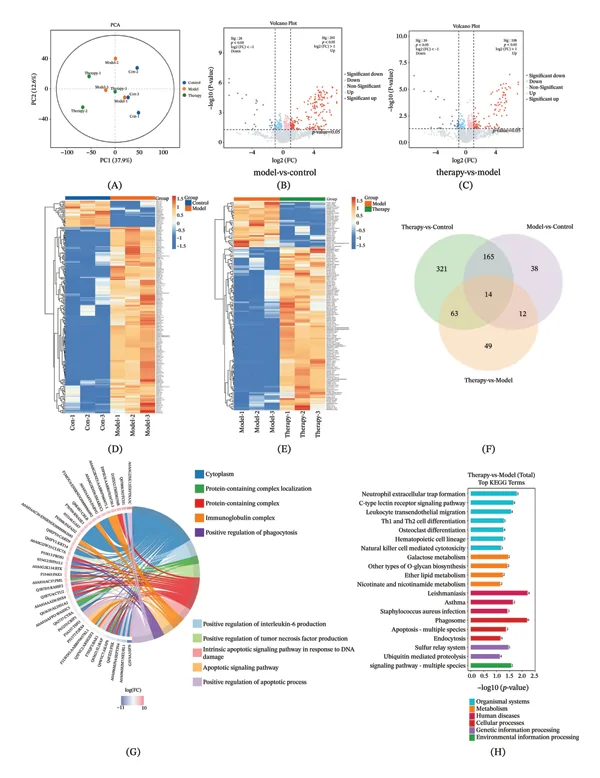
Proteomics analysis of muscle tissue of rats (A) Principal component analysis (PCA) in the Control, Model, and Therapy. (B) Volcano plot of DEPs in the Model and Control groups. (C) Volcano plot of DEPs in the Therapy and Model groups. (D) Heatmap of the expression of DEPs in the Control and Model groups. (E) Heatmap of the expression of DEPs in the Model and Therapy groups. (F) Venn diagram showing the shared and unique identified proteins among the Control, Model, and Therapy groups. DEPs, differentially expressed proteins. (G) Chord diagram illustrating enriched GO terms. (H) KEGG enrichment analysis of differential proteins.

Three samples were selected from each group for proteomic analysis of injured muscle tissue. Based on DIA proteomics results, PCA was performed using protein expression data to visualize sample relationships across different dimensions. Each point represents one replicate from the grouped experiment, with different colors distinguishing between groups (Figure 7A). PCA revealed sample relationships across various dimensions, with samples from the same group exhibiting greater spatial dispersion. The volcano plots in Figure 7B,C reveal 229 differentially expressed proteins between the Control and Model groups, with 201 showing an upregulation trend and 28 exhibiting a downregulation trend. The Treatment and Model groups revealed 138 differentially expressed proteins, with 108 showing an upregulation trend and 30 exhibiting a downregulation trend. The Venn diagram indicates that all differentially expressed proteins met the significance threshold of *p* < 0.05. Figure 7D,E presents a clustering heatmap visually illustrating differentially expressed proteins across the three tissue groups. These proteins exhibited dynamic changes among the groups. KEGG enrichment analysis of the Treatment and Model groups revealed key signaling pathways involved, including neutrophil extracellular trap formation, C‐type lectin receptor signaling pathway, leishmaniasis, phagosome, and endocytosis.

### 3.7. Metabolomic Analysis of Manipulative Therapy on Damaged Muscles in Rats With CLBP

**FIGURE 8:**
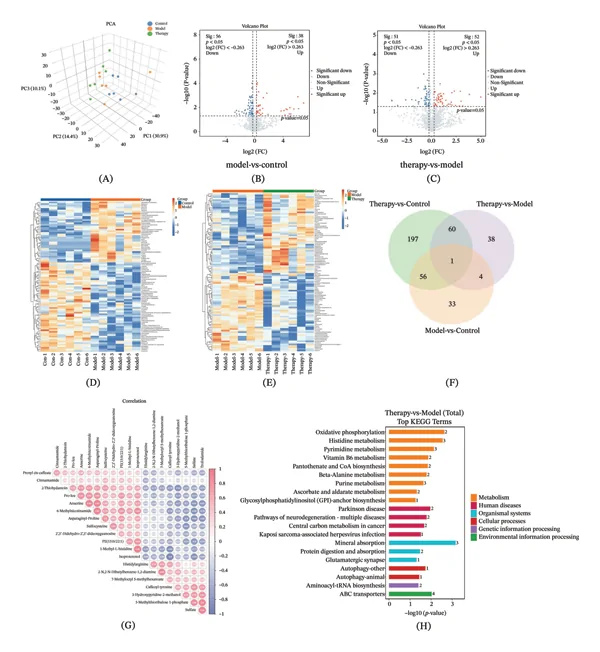
Metabolomics analysis of muscle tissue of rats. (A) Principal component analysis (PCA) in the Control, Model, and Therapy. (B) Volcano plot of DEMs in the Model and Control groups. (C) Volcano plot of DEMs in the Therapy and Model groups. (D) Heatmap of the expression of DEMs in the Control and Model groups. (E) Heatmap of the expression of DEMs in the Model and Therapy groups. (F) Venn diagram showing the shared and unique identified metabolites among the Control, Model, and Therapy groups. DEMs, differentially expressed metabolites. (G) Pearson’s correlation coefficient analysis shows the metabolic network among significantly different metabolites. (H) KEGG enrichment analysis of metabolites.

First, PCA was performed to assess the stability of the system. Figure 8A indicates that the experiment was stable and reproducible. The volcano plot revealed significant differences in metabolites between groups. Compared with the Model group, the Control group showed 94 differentially expressed metabolites, including 38 upregulated and 56 downregulated metabolites. Compared to the Model group, the Treatment group exhibited 103 differentially expressed metabolites, including 52 upregulated and 51 downregulated metabolites (Figure 8B,C). The clustering heatmap displayed the dynamic changes of differential metabolites across groups (Figure 8F,G). The Venn diagram showed five common differential metabolites between the Model group vs. Control group and the Model group vs. Treatment group (13‐amino‐tridecanoic acid, erythrose, hexaethylene glycol, N,N‐dimethyldodecylamine, sulfocysteine) (Figure 8F). Correlation analysis revealed associations among different metabolites (Figure 8G). KEGG pathway enrichment analysis identified significantly enriched differential pathways including key signaling cascades such as mineral absorption, oxidative phosphorylation, histidine metabolism, and pyrimidine metabolism (Figure 8H).

### 3.8. Integrated Multi‐Omics Analysis

**FIGURE 9:**
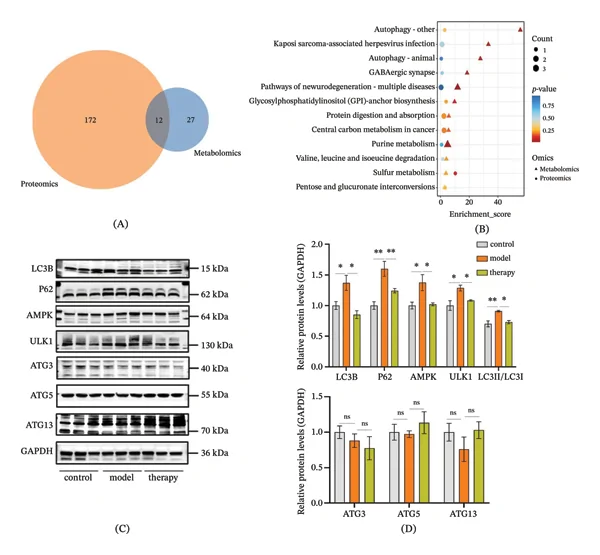
Integrated multi‐omics analysis indicates potential involvement of the AMPK/ULK1‐associated autophagy cascade in MT intervention. (A) Venn diagram illustrating the intersection of signaling pathways enriched by both differentially expressed proteins (DEPs) and differentially expressed metabolites (DEMs). (B) Bubble plot displaying the top 10 shared KEGG pathways, highlighting the significant enrichment of the autophagy signaling pathway. (C) Representative Western blot bands for AMPK, ULK1, LC3B, p62/SQSTM1, and downstream autophagy markers (ATG3, ATG5, ATG13) in the multifidus muscle. GAPDH was used as a loading control. (D) Densitometric quantification of relative protein expression levels normalized to GAPDH. Statistical comparisons were performed via one‐way ANOVA followed by Tukey’s post hoc test. Data are presented as mean ± SD (*n* = 3). ^∗^ *p* < 0.05, ^∗∗^ *p* < 0.01, ^∗∗∗^ *p* < 0.001.

Traditional Chinese medical classics state: “Pressure disperses blood and qi, thus alleviating pain upon palpation.” The principle of “external treatment with internal resonance” evolved through generations of physicians’ prolonged practice in understanding and treating diseases. Therefore, we integrated proteomics and metabolomics data from muscle tissue. The Venn diagram revealed 12 signaling pathways: autophagy‐other; Kaposi’s sarcoma‐associated herpesvirus infection; autophagy‐animal; *γ*‐aminobutyric acid synapse; neurodegenerative disease pathways; multiple diseases; glycerophosphoinositol (GPI)‐anchoring biosynthesis; protein digestion and absorption; central carbon metabolism in cancer purine metabolism; valine, leucine, and isoleucine degradation; sulfur metabolism; and pentose‐glucuronic acid interconversion (Figure 9A,B). Among these shared pathways, autophagy signaling is significantly enriched. AMPK, acting as an energy sensor, is activated when cellular energy levels decline. Activated AMPK directly phosphorylates multiple sites on ULK1 (e.g., Ser317, Ser555), promoting autophagy initiation. Given the interaction mechanism between AMPK and autophagy signaling pathways, our multi‐omics intersection data only show that autophagy signaling is perturbed after MT, representing a correlative molecular signature rather than confirmed therapeutic mechanism.

### 3.9. Mechanistic Validation of Mechanotherapy on the AMPK/ULK1‐Mediated Autophagy Pathway

To elucidate the molecular mechanism by which MT modulates autophagic signaling, we employed Western blot analysis to quantify the protein abundance of the energy sensor AMPK, the primary autophagy initiator ULK1, and key autophagic markers, including the lipidated microtubule‐associated protein 1 light chain 3B (LC3B) and the ubiquitin‐binding scaffold protein SQSTM1/p62. Our results demonstrated that the expression levels of AMPK and ULK1 were significantly upregulated in the multifidus muscle of the Model group compared to the Control group (*p* < 0.05), accompanied by a marked increase in LC3B and a concomitant accumulation of SQSTM1/p62. This pattern suggests that CFA‐induced chronic inflammation triggers aberrant autophagic activation or potentially induces autophagic stress characterized by impaired lysosomal clearance. Notably, MT interventions significantly reduced the overexpression of AMPK and LC3B (*p* *< 0.05*). The LC3II/LC3I ratio was also decreased following treatment. Both ULK1 and p62 showed consistent downward trends toward baseline levels (*p* *< 0.05*). To further delineate the specific stage of the autophagic cascade targeted by MT, we expanded our validation to include essential downstream elongation and maturation components, specifically ATG3, ATG5, and ATG13. Interestingly, no statistically significant differences in the expression of these proteins were observed between the Control, Model, and Treatment groups (*p* *> 0.05*). Taken together, these data only demonstrate correlative changes of AMPK/ULK1 autophagy markers after MT intervention; causal regulatory relationships of this signaling cascade need further functional verification.

## 4. Discussion

MT achieves bottom‐up conduction by altering the microenvironment within peripheral injury sites through pressure applied to cutaneous and intramuscular receptors in localized areas, thereby maximizing the restoration of structure and function within the sensorimotor neural circuit. MT has been practiced in China for many years and shares certain similarities with acupuncture. According to the Global Burden of Disease study, low back pain has consistently ranked among the leading causes of years lived with disability (YLDs) worldwide [42, 43]. The lumbar region is susceptible to various stressors, and prolonged irritation can develop into CLBP [44]. Soft tissue pain may be the primary cause of CLBP [45]. This study induced muscle inflammation in rats by injecting CFA into the multifidus muscle. MPWT testing and the OFT revealed that MT significantly improved pain‐related behaviors and enhanced locomotor activity in the Treatment group.

PFC is a complex brain region that regulates a wide range of functions, from cognition, emotion, and executive behavior to even pain processing [46]. Patients with CLBP exhibit reduced activation in the anterior cingulate cortex, PFC, and nucleus accumbens [47]. This study employs proteomics and metabolomics analysis of the PFC, which governs damaged muscle and pain processing, to reveal the molecular mechanisms underlying MT’s efficacy in alleviating CLBP.

Pain is estimated to affect over 20% of the global population, imposing an immeasurable burden on health and the economy. Effective pain management is crucial for individuals suffering from pain [48]. CFA not only induces inflammation at the injection site but also causes pain hypersensitivity, elevated body temperature, and significantly reduced locomotor activity at sites distant from the injection site [49].

This study employed moderate‐intensity MT to alleviate CLBP, with the intensity and frequency demonstrated to be effective across multiple studies [50]. MT can promote muscle tissue repair, improve fibrosis levels, and reduce the expression of inflammatory cytokines IL‐6 and IL‐1β. Researchers have established a model of gastrocnemius muscle injury using CTX, finding that mechanical stimulation promotes the recovery of muscle tissue [51]. Our results are consistent with those of previous studies.

A pivotal finding of this study is the modulation of the autophagy signaling pathway in the multifidus muscle. Autophagy acts as a double‐edged sword; while basal autophagy is homeostatic, excessive or impaired autophagy contributes to cell death and tissue damage. In our CFA‐induced model, we observed significantly elevated levels of AMPK, ULK1, and LC3B, accompanied by the accumulation of p62 (SQSTM1). The concurrent upregulation of LC3B (autophagosome formation) and p62 (substrate accumulation) indicates a state of impaired autophagic flux, where the degradation machinery fails to keep pace with induction, leading to cellular stress. This upregulation of AMPK likely represents a compensatory but futile response to metabolic stress caused by chronic inflammation. Strikingly, MT significantly attenuated the aberrant elevation of these proteins compared to the Model group, although they did not fully return to baseline levels, suggesting partial rather than complete reversal of the autophagic response. This reduction does not imply the inhibition of necessary physiological autophagy, but rather the restoration of autophagic flux balance and the resolution of cellular stress. By improving the inflammatory microenvironment and reducing metabolic demand, MT correlates with reduced abnormally elevated AMP expression triggered by chronic inflammatory stress. This mechanism aligns with recent studies suggesting that restoring autophagic homeostasis rather than simply boosting or blocking it is key to tissue recovery in chronic inflammatory conditions.

**FIGURE 10:**
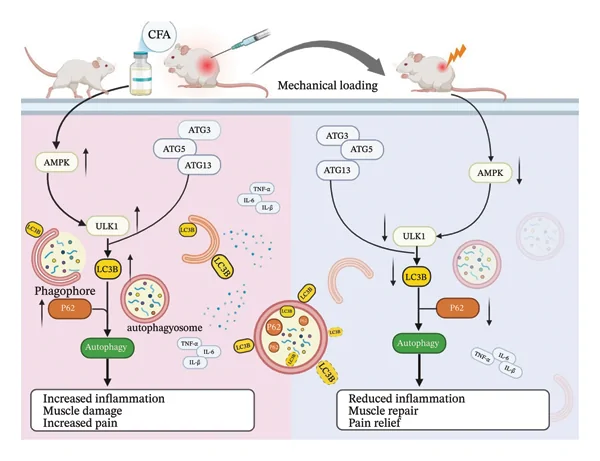
Schematic of correlative molecular changes after MT in CFA‐induced inflammatory low back pain rats; causal pathway validation is needed.

Protein levels of LC3B and p62 in rats in the Treatment group were lower than those in the Model group. MT may alleviate inflammation‐triggered aberrant autophagic accumulation, with the AMPK/ULK1 cascade potentially participating in this regulatory process (Figure 10). Autophagy is an important metabolic process within cells that helps maintain homeostasis, differentiation, development, and survival. Autophagy is considered a highly selective cellular clearance pathway associated with maintaining cellular homeostasis. It is one of the key mechanisms coordinating the differentiation and metabolic state of innate immune cells. The levels and balance of different forms of autophagy in each organism are highly specific at every stage of life [52]. Maintaining autophagy balance within the body is a challenge [53]. This study demonstrates that MT may promote health by improving the inflammatory microenvironment and balancing autophagy.

This study has several limitations. Research on the quantification and standardization of techniques presents challenges. Previous studies have applied a pressure of 3–5 N. We selected 4 N pressure, delivered via a massage head targeting the injured region, based on published literature and our preliminary tests. This approach achieved therapeutic effects without causing obvious aversion to MT. Multiple brain regions collectively process pain information within the brain. This study primarily focuses on the PFC and cannot comprehensively analyze the central mechanisms of MT for pain relief. Although this study explored mechanisms based on information from proteomics and metabolomics, it did not employ pathway agonists or inhibitors for validation. Notably, the present immunoblot measurements of static LC3B and p62 abundance cannot reliably reflect autophagic flux dynamics. Quantitative assessment of autophagic turnover, specific detection of phosphorylated AMPK and ULK1, as well as pharmacological or genetic perturbation of the AMPK/ULK1 cascade are required to rigorously verify pathway activity and establish causal relationships. Beyond this, organ–organ interactions have emerged as a research hotspot in recent years, with brain–muscle interactions validated across multiple diseases [54, 55]. Whether MT modulates brain–muscle signaling communication during chronic pain and the underlying regulatory pathways remain to be further elucidated.

## 5. Conclusion

In conclusion, our integrated multi‐omics and molecular data show that MT alleviates muscle inflammation and alters AMPK/ULK1‐associated autophagy protein levels in CFA‐induced rats. These correlative molecular changes cannot confirm a direct causal pathway, and further functional validation is required. These preclinical findings uncover a mechanistic pathway of MT in CFA‐induced inflammatory multifidus injury, generating testable hypotheses about peripheral tissue remodeling and central metabolic plasticity. Further research using diverse CLBP animal models and human cohorts is essential to verify whether this regulatory axis can be translated to the heterogeneous spectrum of clinical CLBP.

## Author Contributions

Liangyu Xie and Zuozhen Yin: writing–original draft, visualization, software, methodology, formal analysis, and data curation. Litao Shao: visualization, software, methodology, and data curation. Jiale Ma: methodology, investigation, formal analysis, and data curation. Haowen Cheng: methodology, formal analysis, and data curation. Liang Shi and Gongchang Yu: data curation. Shengnan Cao, Weidong Su, and Bin Shi: supervision, resources, project administration, investigation, and funding acquisition.

## Funding

This work was supported by National Natural Science Foundation of China (82374615, 82405600), the clinical‐basic innovation team project of Shandong First Medical University (CX202408), the Key Research and Development Program of Shandong Province (Competitive innovation platform) (2026CXPT243), the Joint‐constructed Science and Technology Project of the Department of Science and Technology, National Administration of Traditional Chinese Medicine (GZY‐KJS‐SD‐2023‐082), Taishan Scholars Youth Experts Program (tsqn202507377), and Shandong Provincial Medical and Health Development Program (202404070353).

## Ethics Statement

The animal experiment was authorized by the Animal Ethics Committee of Neck‐Shoulder and Lumbocrural Pain Hospital of Shandong First Medical University and carried out in compliance with applicable regional laws and institutional standards.

## Conflicts of Interest

The authors declare no conflicts of interest.

## Data Availability

The raw proteomics and metabolomics datasets generated in this study have been deposited in the Genome Sequence Archive (GSA) at the National Genomics Data Center (https://ngdc.cncb.ac.cn/gsa) under the accession number PRJCA070093. Processed analytical data, uncropped Western blot images, and plotting source files are available from the corresponding authors upon reasonable request.
